# IKAP model-based nursing interventions on elderly patients with chronic obstructive pulmonary disease and its impact on self-management ability and quality of life

**DOI:** 10.4314/ahs.v25i3.19

**Published:** 2025-09

**Authors:** Sha Lv, Yueju Du, Zhiping Sun, Xiufeng Tian

**Affiliations:** 1 The First Department of Respiratory and Critical Care Medicine, Hebei Chest Hospital, Shijiazhuang, Hebei, 050000; 2 Department of Clinical Laboratory, Hebei Chest Hospital, Shijiazhuang, Hebei, 050000; 3 The Second Department of Respiratory and Critical Care Medicine, Hebei Chest Hospital, Shijiazhuang, Hebei, 050000

**Keywords:** IKAP, COPD, Self-management ability, Cardiopulmonary function

## Abstract

**Objective:**

To study the effect of information-knowledge-belief-behavior (IKAP) model-based nursing interventions on elderly patients with chronic obstructive pulmonary disease (COPD)and its impact on self-management ability and quality of life.

**Methods:**

During January 2021 to December 2021, 88 elderly patients with stable COPD cured in our hospital were arbitrarily classified into research group (n=44) and control group (n=44). The former accepted nursing intervention based on IKAP model, while the latter accepted routine nursing. The scores of Morisky medication compliance scale, self-management ability, modified British Medical Research Council modified Respiratory Disorder scale (mMRC), 6-minute walk test and life quality assessment questionnaire of COPD patients were studied before and after 4 weeks of nursing. The data were processed with SPSS23.0 statistical software.

**Results:**

According to the Morisky drug compliance scale, the research group scored 7.25 after 4 weeks compared to the control group's score of 6.18 (P<0.05). Research group members scored 181.19 more on self-management ability following 4-week nursing than control group members scored 159.15. Research group 1.72 had a lower mMRC scale score after 4 weeks of nursing than control group 2.15 (P<0.05). Following 4-week care, the research group's 6-minute walking test value was 351.23, compared with the control group's 318.44 (P<0.05). Researchers found that after 4 weeks of nursing, COPD patients in the research group scored 19.47 on the quality of life assessment questionnaire, compared with 24.68 in the control group (P<0.05).

**Conclusion:**

The clinic application value of nursing intervention based on IKAP model in elderly patients with stable COPD is remarkable, which is beneficial to enhance patients' compliance with medication, ability of self-management, and lessen the condition of dyspnea.

## Introduction

As a result of progressive airflow obstruction caused by chronic obstructive pulmonary disease (COPD), the disease suffers from high morbidity, mortality, and disability, which has become an important public health problem^1^. Some studies have shown that China is gradually stepping into the social background of aging and the number of COPD patients is showing a trend of increasing year by year^2^. According to the global burden of disease (GBD) research statistics, the death and disability rate of COPD is the fifth economic burden of disease in the world, ranking the third cause of death^3^. In China, the number of COPD patients is about 100 million. A prevalence rate of 27% of COPD has been found among people older than 60 years old^4^. At present, COPD treatment mainly consists of acute exacerbation treatment and stable rehabilitation. The main treatment strategy is to control the symptoms of patients and delay the development of the disease through drug therapy and non-drug therapy^5^. It has increased and seriously threatens the life and health of elderly COPD patients^6^,^7^. Some studies have shown that self-management programs play a role in promoting long-term disease management in patients with COPD^8^,^9^. To change patients' attitudes and behaviors by increasing their knowledge about the subject. To help patients improve self-care behaviors, cope effectively with symptoms, improve compliance and better reduce the burden on patients and society^10^,^11^. Airflow limitation and persistent respiratory symptoms are indicators of COPD, an airway disease that is one of the most common chronic diseases. The incidence of COPD is high in the elderly. COPD will not only have serious adverse effects on the life quality of patients, but also lead to serious consequences such as death of patients.

Information-knowledge-attitude-practice (IKAP) model is a kind of health-related behavior change model extended by domestic scholars based on KAP (Knowledge-attitude-practice) theory, which was first proposed by Rosalie at the end of 20th century^12^-^13^. The KAP model of care combined with individual dietary modification could help to manage the condition of patients with COPD type 2 diabetes, improve their confidence in coping with the disease and reduce their fear of disease progression^14^. Research confirms that there is a causal relationship among knowledge, attitude, belief and behavior. Only by accumulating sufficient knowledge can positive beliefs and attitudes be formed, thus promoting behavior change^13^-^14^. Furthermore, many studies have confirmed that health guidance or nursing programs based on IKAP model can improve chronic disease patients' disease awareness, enhance their self-efficacy, promote their compliance with doctor's advice, alleviate their psychological state, and enhance their life quality^15^-^17^. Li et al. found that continuous nursing based on IKAP theory improved the score quality of COPD patients 3 months after intervention^18^. The research of Zhang Yingdi et al. indicated that continuous health education based on IKAP theory could promote the life quality, satisfaction and self-efficacy of COPD patients^19^. Jing Xuan found that nursing intervention based on IKAP model can enhance the self-management ability and self-efficacy of COPD patients, and reduce the influence of disease on patients^20^. Although IKAP model has been widely adopted in many specialist fields of chronic diseases, containing cardiovascular and cerebrovascular diseases, cancer, metabolic diseases, chronic respiratory diseases. Patients with chronic respiratory diseases rarely use it to self-manage their condition. The research innovation of this paper was this study used the IKAP model to construct a self-management intervention program that is consistent with the stable stage of COPD in elderly patients in China and to validate its effectiveness.

## Patients and methods

### General information

During January 2021 to December 2021, 88 elderly patients with stable COPD cured hospitalized were arbitrarily classified into research group (n=44) and control group (n=44). In the former group, there were 22 men and 22 women, who aged from 61 to 79 years with an average of 68.48±3.28 years. The course of disease ranged from 3 to 7 years (5.29 ±2.11). In the latter group, there were 21 men and 23 women, who aged from 60 to 78 years with an average of 68.15±3.31 years. The duration of the disease ranged from 3 to 7 years (5.32± 2.07). Data in general did not exhibit a statistically remarkable difference (P>0.05).

**Inclusion criteria:** (1) age ≥ 60 years old; (2) the patients met the diagnostic criteria of guidelines to diagnose and treat COPD (revised in 2013)^21^; (3) Grade II-III of pulmonary function; (4) stable condition and clear consciousness; (5) Study participants and their families signed informed consent forms after receiving information about the study.

**Exclusion criteria:** (1) patients with severe chronic diseases, such as malignant tumors, active pulmonary tuberculosis or other fatal diseases; (2) patients with a history of stroke or dementia; (3) patients with severe limb dysfunction, such as paralysis, amputation, etc.; (4) patients with long-term use of drugs that may cause anxiety and depression. (5) the patients were unable to complete the scale due to severe aphasia, disturbance of consciousness, lack of comprehension, mental illness or other reasons.

## Treatment methods

### Technical route

#### Intervention scheme

Schemes in the control group received routine care. The contents of routine care include: (1) Explain the common causes, symptoms, treatment principles and nursing measures of the disease to patients; (2) Introduce the role of drugs, adverse reactions, and precautions to patients; (3) Inform patients about the importance of obeying doctor's orders sex. All patients received 4 weeks of usual care.

Research group plan: Nursing intervention based on IKAP mode was carried out. (1) Information (I). Within 24 hours after admission, the patients filled in the personal information form, self-management needs questionnaire and related questionnaires. The patients' basic personal information was evaluated, such as physical status, mental status, family income, as well as self-management needs information, such as disease knowledge, medication guidance, smoking cessation guidance, exercise guidance, psychological guidance, nutritional guidance. According to patient information, self-management intervention program was refined and carried out on the day before and on the day before discharge. 2) Knowledge (K). 1) COPD related knowledge: epidemic characteristics, disease symptoms, signs, inducements, risk factors, diagnostic methods, treatment principles, complications, prognosis and harm. 2) Knowledge and skills of rehabilitation exercises: clarification of the importance and purpose of exercises such as lung function; methods of ventilator exercises: methods, timing and frequency of labial contraction breathing and abdominal breathing, upper and lower limb exercises, etc.; effective methods of coughing and sputum evacuation. 3) medication knowledge and skills were the efficacy and adverse reactions of drugs, the importance of following doctor's orders, the effects of rational drug use on the disease and the harm of self-withdrawal of drugs to the deterioration of the disease. And medicine kits marked “early-middle-late” were distributed at the same time. Correct and effective use of inhalers and equipment (such as dispensers, Dubao devices, powder inhalation dispensers). 4) Quitting education included the harm of tobacco, the effect of smoking on lung diseases, the change of human function in different days of quitting smoking, and the methods of quitting smoking. 5) knowledge and methods of disease prevention: environment, diet, activity and rest, exercise, going out and other precautions and specific methods; methods of preventing infection. 6) pay attention to the indication of medical treatment and the treatment of acute attack: fever, shortness of breath or wheezing, aggravation of cough, increase of sputum, change of sputum color, change of sputum viscosity and so on. 7) reasonable dietary knowledge and methods: high protein, high vitamin, easy to digest and low salt diet, avoid spicy stimulation, gas-producing food, drink more water, avoid smoking and drinking, etc. (3) Belief (A). 1) case sharing: the correlation between self-management success cases, failure cases and disease prognosis and life quality, and encourage patients to express their feelings. 2) experience exchange (peer education): carry out patient symposia, exchange and share the experience of disease self-management, or share the insights through micro-video. 3) personal talk: patients are encouraged to reflect on themselves, at least three reasons that hinder self-management are mentioned, and the root causes are traced back with professionals to solve them. (4) Behavior (P). 1) push knowledge through WeChat official account: push disease management-related knowledge, breathing training and other skills taught to patients to the official account in the form of easy-to-understand pictures and texts or short videos, and set up patient reminders. 2) establish WeChat management group of patients: on the day of discharge, scan the code to enter the “self-management group of elderly COPD patients”. After discharge, WeChat group was used for management, including sign-in, supervision, education, communication, daily reports, weekly summary and online consultation.

#### Observation index

(1) To study the 6-minute walking test (6MWT) before nursing and after 4 weeks of nursing^22^. In this study, the test site was selected in the 30-meter corridor of the respiratory department, telling patients to wear comfortable clothes and shoes during the test. Oxygen inhalation was stopped half an hour before the experiment and rested for more than 10 minutes. Measurements of blood pressure, heart rate, respiratory rate, finger pulse oxygen, and fatigue and dyspnea were performed at the beginning of the test. During the experiment, patients were required to do their best to walk quickly within 6 minutes. The experiment was conducted twice. The longest walking distance of the patient was selected as the experimental results. All kinds of first aid items and equipment should be prepared in the testing process.
(2) The self-management ability scores before nursing and 4 weeks after nursing were studied. The table adopts the COPD patient self-management scale compiled by domestic scholars Zhang Caihong et al.^23^, which included 51 symptom management, daily life management, information management, emotional management and self-efficacy. Less than 124 score was low level; 124 to 180 score was middle level; more than 180 score was high level with good internal consistency reliability (Cronbach's α = 0.92).
(3) In this study, the degree of dyspnea was evaluated by the modified British Medical Research Council's modified dyspnea scale (mMRC)^24^. The scale was based on the subjective feelings of the patients, the degree of dyspnea was classified into 0-4.
(4) To determine the score on the Morisky Drug Compliance Scale (MMAS) before nursing as well as 4 weeks after nursing. The scale included a total of 8 questions, which are scored by the assigned scoring system. The total score < 6 was regarded as low compliance, the total score ≥ 6 and < 8 as moderate compliance. The total score = 8 as high compliance. The Cronbach's α coefficient of the scale was 0.83^25^. The reliability and validity of the Morisky Medication Adherence Scale were high. The scale can reflect Medication Adherence in patients.
(5) To study the questionnaire score of quality-of-life assessment of COPD patients before nursing and 4 weeks after nursing. This study used the COPD Assessment questionnaire (CAT)^26^, which could accurately reflect the severity of clinical symptoms and the influence of life quality in patients with COPD. The scale contained 8 items, such as cough, expectoration, chest tightness, activity, ability of daily living, ability to go out, sleep quality and energy. A total score of 0-5 was calculated for each question according to the 6-point scoring system. The total score of the questionnaire was 0-40, which was inversely proportional to the life quality, in which 0-10 was slightly affected, 11-20 was moderately affected, 21-30 was seriously affected, and 31-40 was extremely seriously affected.

### Statistical analysis

Data analysis was performed using SPSS23.0 software. The measurement data were expressed as (±s). An analysis of variance was used to compare multiple groups, as well as a group design t-test for the comparison between two groups. Comparison with the control group was conducted using the Dun-net-T test. In all statistical tests, the data were presented as the number of cases and the percentage, a χ2 test was used to compare groups, and a bilateral test was used for all comparisons outside of the study group.

## Results

### The scores of Morisky drug compliance scale before nursing and 4 weeks after nursing

Compared to the control group, the research group had a higher Morisky drug compliance score after four weeks of nursing (P<0.05, Table 1).

**Table 1:** The scores of Morisky drug compliance scale before and after 4 weeks of nursing

Grouping	Before nursing	After 4 weeks of nursing
Control group	4.12±1.08	6.18±0.46*
Research group	4.14±1.11	7.25±0.14*
*t value*	0.086	14.761
*P value*	0.932	<0.01

Note:

* represents the comparison of this group before nursing and 4 weeks after nursing, P<0.05

### The scores of self-management ability before nursing and after 4 weeks of nursing

In the research group, self-management ability improved after four weeks of nursing compared with the control group (P<0.05, Table 2).

**Table 2:** The scores of self-management ability before nursing and after 4 weeks of nursing

Grouping	Before nursing (points)	After 4 weeks of nursing (points)
Control group	115.28±10.44	159.15±13.27*
Research group	115.31±10.37	181.19±15.13*

*t value*	0.014	7.264
*P value*	0.989	<0.01

Note:

* represents the comparison of this group before nursing and 4 weeks after nursing, P<0.05

### The score of mMRC scale before nursing and 4 weeks after nursing

According to the research group, the score of the mMRC scale was lower after 4 weeks of nursing than in the control group (P<0.05, Table 3).

**Table 3:** The score of mMRC scale before nursing and 4 weeks after nursing

Grouping	Before nursing (points)	After 4 weeks of nursing (points)
Control group	3.38±0.23	2.15±0.19*
Research group	3.41±0.21	1.72±0.03*
*t value*	0.639	14.828
*P value*	0.525	<0.01

Note:

* represents the comparison of this group before nursing and 4 weeks after nursing, P<0.05

### The 6-minute walking test before nursing and 4 weeks after nursing

In the research group, the 6-minute walking test was higher after 4 weeks of nursing than in the control group (P<0.05, Table 4).

**Table 4:** The 6-minute walking test before nursing and 4 weeks after nursing

Grouping	Before nursing	After 4 weeks of nursing
Control group	208.31±9.31	318.44±10.25*
Research group	208.42±9.25	351.23±12.47*
*t value*	0.059	13.474
*P value*	0.953	<0.01

Note:

* represents the comparison of this group before nursing and 4 weeks after nursing, P<0.05

### The score of COPD life quality assessment questionnaire before and 4 weeks after nursing

COPD life quality assessment questionnaire scores did not differ remarkably before and after nursing (P>0.05). During the first 4 weeks of nursing, the research group's COPD life quality assessment scores were lower (P<0.05, Table 5).

**Table 5:** The scores of COPD life quality assessment questionnaire before and 4 weeks after nursing

Grouping	Before nursing	After 4 weeks of nursing
Control group	33.36±4.17	24.68±3.43*
Research group	33.43±4.25	19.47±1.25*
*t value*	0.078	9.467
*P value*	0.938	<0.01

Note:

* represents the comparison of this group before nursing and 4 weeks after nursing, P<0.05

## Discussion

Inpatients with acutely exacerbated COPD reached a stable period after treatment and returned home to recover on their own. After discharge from hospital, most patients lack effective long-term ongoing management guidance from the hospital. There is also a lack of standardized guidance on chronic obstructive pulmonary care in the primary community, leading to an increase in the number of acute exacerbations in patients with COPD^27^,^28^. To date, there is no complete cure for COPD in the world. Regulated treatment can control the disease, and the effectiveness of treatment depends on patient compliance with health behaviors. The Global Initiative for Chronic Obstructive Lung Disease (GOLD) states that patient education, medication monitoring and regular follow-up attach importance to improving patient compliance, reducing acute exacerbations, improving patients' life quality and reducing healthcare costs^29^. Older people, the main group of patients with COPD, may suffer from varying degrees of frailty and cognitive impairment. Older patients are unable to complete rehabilitation programmes as required, resulting in poor disease recovery outcomes^30^,^31^. Therefore, the management and guidance of elderly patients with stable COPD should be strengthened so that they can truly benefit from it. This study used the IKAP model to construct a self-management intervention program that is consistent with the stable stage of COPD in elderly patients in China and to validate its effectiveness.

Self-management is defined as a health-promoting approach, often used to cope with chronic diseases^32^. For COPD patients, effective self-management is critical for promoting healthy behaviors, relieving disease symptoms. The IKAP model is the localization theoretical achievement of domestic scholars based on the KAP theory, which emerged at the end of the 20^th^ century. From the results of its application, the IKAP model-based nursing intervention has been shown to have a remarkable effect on improving disease management and life quality in patients with chronic diseases. It is of great significance in complying with medical practices, achieving the total management of chronic diseases and reducing the burden of disease^33^.

Self-efficacy is capability of attaching importance to maintaining motivation and goal-directed behavior^34^-^35^, which is consistent with outcomes in the IKAP model, emphasizing that personal beliefs and attitudes are key factors in self-management behavior, and support individuals to persevere in accomplishing goals and related powerful motivation for behavior. The results indicated that after the nursing intervention based on the IKAP model, the scores of Morisky medication compliance scale, self-management ability score, and 6-minute walk test values of patients were all higher compared to routine nursing. Comparing routine care to mMRC scale and COPD life quality assessment questionnaire, the score of the control group was higher. The IKAP model-based nursing intervention in elderly stable COPD patients has proven to be beneficial in improving patients' medication compliance, self-management and cardiopulmonary function. This is mainly because, self-efficacy affects individuals' adherence to health-promoting behaviors, thereby improving physical function in chronic disease management theory^34^-^35^. In the first stage, general information and medical history is obtained and captured through observation, communication, questioning, physical examination and review of medical records. The center conducts self-management interventions with full knowledge of the patient's basic information, covering modules such as knowledge inculcation, skills transfer, belief enhancement and behavioral transformation^36^. In the prevention phase, medical concepts should be changed and health education should be integrated into all aspects of patient self-management. Patients improve their sense of self-efficacy and can take their medication as prescribed, exercise on time, eat properly, prevent disease progression and master countermeasures for disease progression. In the COPD phase, patients consciously adhere to the correct self-management behaviours, thus improving the symptoms of dyspnoea and reducing the impact of the disease on the patient^37^, ^38^. This study still has some shortcomings. Firstly, the quality of this study is limited due to the small sample size we included in the study. Secondl, this research is a single-center study and our findings are subject to some degree of bias. Therefore, our results may differ from those of large-scale multicenter studies from other academic institutes. This research is still clinically significant and further in-depth investigations will be carried out in the future.

## Conclusion

In elderly patients with stable COPD, nursing interventions based on the IKAP model have a greater application value. The clinic application value of nursing intervention based on IKAP model in elderly patients with stable COPD is remarkable, which is beneficial to enhance patients' compliance with medication, ability of self-management, and lessen the condition of dyspnea.

## Figures and Tables

**Fig. 1:**
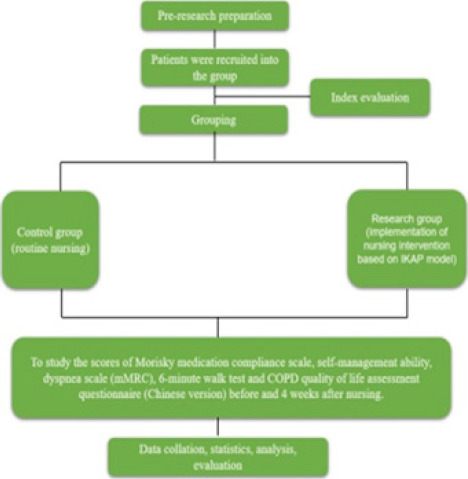
Technology Roadmap

## Clinical Registration Number

ChiCTR2100045100.
